# Role of Beta-Carotene in Lung Cancer Primary Chemoprevention: A Systematic Review with Meta-Analysis and Meta-Regression

**DOI:** 10.3390/nu14071361

**Published:** 2022-03-24

**Authors:** Jacek Kordiak, Filip Bielec, Sławomir Jabłoński, Dorota Pastuszak-Lewandoska

**Affiliations:** 1 Department of Thoracic, General and Oncological Surgery, Medical University of Lodz, 90-151 Lodz, Poland; jacek.kordiak@umed.lodz.pl (J.K.); slawomir.jablonski@umed.lodz.pl (S.J.); 2 Department of Microbiology and Laboratory Medical Immunology, Medical University of Lodz, 90-151 Lodz, Poland; dorota.pastuszak-lewandoska@umed.lodz.pl

**Keywords:** beta-carotene, lung cancer, prophylaxis

## Abstract

Lung cancer is one of the most common neoplasms globally, with about 2.2 million new cases and 1.8 million deaths annually. Although the most important factor in reducing lung cancer risk is lifestyle change, most patients favour the use of supplements, for example, rather than quitting smoking or following a healthy diet. To better understand the efficacy of such interventions, a systematic review was performed of data from randomized controlled trials concerning the influence of beta-carotene supplementation on lung cancer risk in subjects with no lung cancer before the intervention. The search corpus comprised a number of databases and eight studies involving 167,141 participants, published by November 2021. The findings indicate that beta-carotene supplementation was associated with an increased risk of lung cancer (RR = 1.16, 95% CI = 1.06–1.26). This effect was even more noticeable among smokers and asbestos workers (RR = 1.21, 95% CI = 1.08–1.35) and non-medics (RR = 1.18, 95% CI = 1.07–1.29). A meta-regression found no relationship between the beta-carotene supplementation dose and the size of the negative effect associated with lung cancer risk. Our findings indicate that beta-carotene supplementation has no effect on lung cancer risk. Moreover, when used as the primary chemoprevention, beta-carotene may, in fact, increase the risk of lung cancer.

## 1. Introduction

Lung cancer remains a neoplastic disease with one of the highest morbidity rates worldwide among both women and men [1,2,3]. According to the GLOBOCAN 2020 study, lung cancer accounted for 11.4% of all neoplasms [3]. Unfortunately, lung cancer cases are diagnosed relatively late as noticeable clinical symptoms only appear in the later stages of cancer development [2]. The median age of patients with a primary diagnosis of lung cancer is 70 years [1], in which case the disease is at an advanced stage or has metastasized [2]. The 5-year survival rate for lung cancer, in general, is 19%, and, depending on the histological type, it may be as high as 25% in the case of non-small-cell lung cancer [1].

In the last decade, the incidence of lung cancer has increased significantly, especially in developing countries. This increase is related to the prevalence of risk factors in these regions, mainly smoking and occupational exposure to dust (including asbestos or vinyl chloride) [2]. With about two-thirds of the lung cancer deaths worldwide attributable to these risk factors, the lung cancer incidence can be effectively reduced through legal solutions and proper education [3].

The most important factor in reducing the risk of lung cancer, and, hence, mortality, is a lifestyle change. To enjoy a long and healthy life without lung cancer, the current recommendations include avoiding smoking, or quitting, avoiding exposure to dust or radiation, and following a healthy diet [1,4]. However, it is more common for higher risk groups to favour more straightforward solutions, such as supplementation with various oral preparations [5].

The use of dietary supplements is increasing worldwide, especially concerning primary and secondary cancer prevention. One of the most frequently supplemented substances is antioxidants, which inhibit the formation of free radicals or catalyse their transformation into inactive derivatives [6]. Free radicals cause many changes in the organism’s redox processes, resulting in damage to proteins and nucleic acids, which leads to cell mutations and carcinogenesis [7,8]. However, many large-scale studies indicate that antioxidant supplementation may itself have adverse effects, increasing the risk of cancer [1,6].

Carotenoids are powerful natural antioxidants capable of scavenging oxygen and chelating the ions involved in the formation of radicals [9]. They can be divided into carotenes and xanthophylls (Table 1). Both groups are similar in structure; however, while xanthophylls contain oxygen atoms, carotenes are purely hydrocarbons [9,10]. Both carotene and xanthophyll serve in plants as accessory pigments, which capture and pass sunlight to chlorophyll a. The main difference that can be seen macroscopically is the colour they give to the plants—from red to yellow; carotenes are responsible for darker colours and xanthophylls for lighter ones. It is related to their ability to absorb the light wave [11].

**Figure 1:**
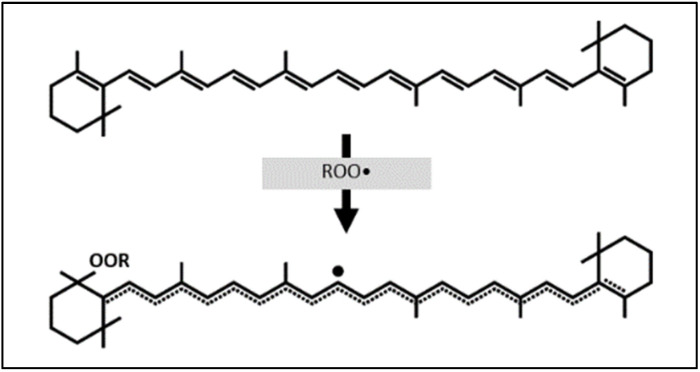
Beta-carotene reaction with free radical (ROO•).

Individual carotenoids have varying antioxidant properties associated with differences in their structures. Carotenes (including the beta-carotene discussed in this systematic review) scavenge free radicals mainly by reducing them, and xanthophylls have better free radical oxidation properties [9]. Beta-carotene is characterized by a high rate of electron acceptance (Figure 1) due to both its ends being cyclized into β-rings [9,11]. However, astaxanthin, a xanthophyll, demonstrates about 50 times greater antioxidant activity than beta-carotene [9]. The ultimate free radical neutralization efficiency of all carotenoids depends on the nature of the free radical, the mechanism of action, and the environment [11].

## 2. Objective

The aim of this systematic review is to provide an overview of the current knowledge regarding beta-carotene supplementation and cancer risk, updating a similar study published more than a decade ago [12]. However, this study focuses solely on lung cancer, being a leading problem among cancer rates. A meta-analysis was performed of studies addressing the effect of beta-carotene intake on the incidence of new lung cancer cases. The results were interpreted with regard to the overall population studied and according to region, sex, beta-carotene dose, medical occupation, and exposure to tobacco smoke or asbestos. A meta-regression was also performed to identify any links between the risk of lung cancer and the dose of supplemented beta-carotene.

## 3. Methods

Two research tools were used: meta-analysis and meta-regression. Meta-analysis is a quantitative synthesis of results from independent research. It provides a synthesis of disparate primary studies, often drawing different conclusions. Meta-regression, however, combines the possibilities of meta-analysis and linear regression and is used to confirm any relationship between the characteristics and the tested effect, providing a deeper insight into the studied variability [13].

### 3.1. Criteria for Considering Studies for Review

Only randomized controlled trials (RCTs) were included in this review, irrespective of their language. The participants of those RCTs were adults (18 years of age or older). The RCTs were considered for inclusion if they were based on beta-carotene supplementation at any dose, frequency, or route of administration, together with a placebo or no intervention group. Trials using beta-carotene in combination with other substances were also included (Table 2). The measured outcome was the incidence of lung cancer, assessed as the proportions of participants developing cancers during the follow-up period.

### 3.2. Search Methods for Identification of Studies

The search was conducted up to November 2021 in the following databases with no restriction on language, publication year, or publication status:

- PubMed (https://pubmed.ncbi.nlm.nih.gov/), (Access on 11 November 2021)
- Web of Science (https://www.webofscience.com/), (Access on 11 November 2021)
- Cochrane Library (https://www.cochranelibrary.com/), (Access on 11 November 2021)
- ClinicalTrials.gov (https://clinicaltrials.gov/). (Access on 11 November 2021)

The following search strategy was used for the PubMed database: “ALL = (((carotene* OR beta-carotene* OR vitamin A) AND (((lung OR pulmonary) AND (cancer OR neoplasm OR carcinoma)) OR (NSCLC OR SCLC)))) AND randomized control* trial.”

There was no restriction on the language. If studies in languages other than English or Polish had been found, the authors sought an initial translation of the abstract to apply the inclusion criteria.

The authors also scanned the references of all relevant articles.

### 3.3. Data Collection

The titles and abstracts identified through the search process were independently reviewed by two authors (J.K. and F.B.). Following this, full texts of the selected articles were retrieved and assessed for eligibility. Any differences of opinion were settled by consensus or referral to other review authors (S.J. or D.P.-L.). There was no blinding of authorship.

The data were extracted again by two review authors (J.K. and F.B.) independently using a standardized data collection form, including article first author, year of publication, name of the study, the total number of participants, a short description of participants, country, age, sex, smoking status, beta-carotene dose, and routine, as well as information about other substances administered, the total number of events, number of events in the intervention group, number of events in the placebo group, follow-up period, and relative risk with their 95% confidence intervals (if available).

### 3.4. Data Analysis

For the conducted meta-analyses and meta-regression, typical risk ratios (RRs) with 95% confidence intervals (CIs) were calculated based on data extracted from articles [14,15,16,17,18]. In the case of two papers [19,20], no exact data on the number of outcomes were found; in this case, the analysis was based on the RRs and 95% CIs taken directly from the articles.

Heterogeneity between studies was tested with a Q-test; the results were considered significant with *p* < 0.05. A fixed-effects model was used in the absence of heterogeneity and a random-effects model in the presence of heterogeneity. All data were analysed using Statistica 13 software (TIBCO Software Inc., Palo Alto, CA, USA).

### 3.5. Limitations

Our systematic review is subject to certain limitations related to the data available for analysis. First, not all RCTs had complete extractable data available. For example, no data on current/former smokers were available for two trials [17,20]. Second, in all trials [14,15,16,17,18,19,20], the intervention consisted of additional antioxidants with beta-carotene. Therefore, the obtained effect may not necessarily depend on supplementation, particularly with beta-carotene; however, the use of meta-analysis should have weakened this disadvantage. Thirdly, the only trial conducted on an Asian population [20] concerned a nutrient-deficient population; the study finding in favour of beta-carotene supplementation could result simply from the compensation of antioxidant deficiencies in the diet and not from any additional supplementation.

## 4. Results

**Figure 2:**
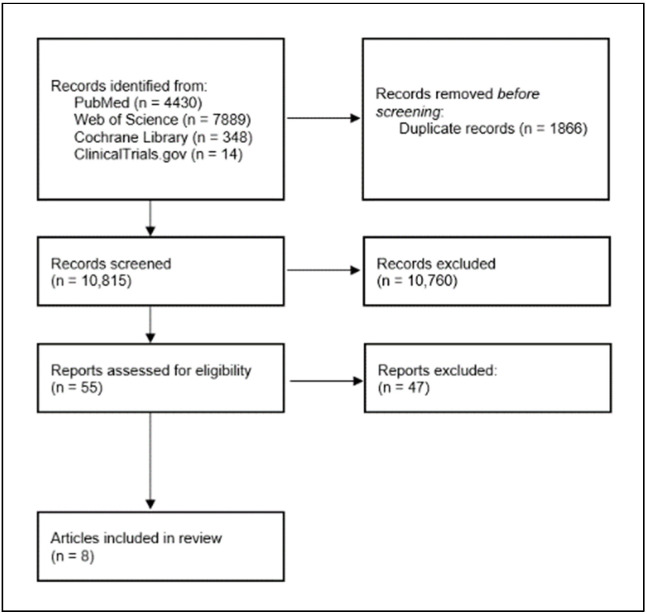
Systematic review flow diagram.

The search results are summarized in the flow diagram in Figure 2. Among the 10,000 publications identified, 55 were potentially appropriate for this review. Forty-seven articles were excluded: 26 due to a wrong research method, 20 due to different intervention, and one due to a different measured outcome. The analysis also included seven publications yielding data on the relationship between lung cancer incidence and beta-carotene supplementation, taken from eight studies.

### 4.1. Characteristics of Included Studies

The characteristics of the studies included in this systematic review are summarized in Table 2. The total number of participants included in all eight studies was 167,141. The study sample size ranged from 4060 [19] to 39,876 [17]. In all the RCTs, the participants were randomly assigned to the intervention or placebo group.

Beta-carotene was supplemented every day in five of the eight trials [14,16,19,20], and on alternate days in the remaining three [15,17,18]. The dose of beta-carotene varied from 15 to 30 mg daily. All the studies assumed the administration of other active substances in addition to beta-carotene [14,15,16,17,18,19,20].

The participants of the trials were diverse. Two studies only included health professionals [15,17], while another two included patients with cardiac risk factors, e.g., cardiovascular disease, diabetes, high serum cholesterol, obesity, or other [16,18]. Two studies included only smokers [14,19], and one study included only asbestos-workers [19]. The remaining trial consisted of a nutrient-inadequate general population [20].

The share of women and men in the reviewed RCTs also differed. Three trials included only men [14,15,19], and another two, only women [17,18]. In the remaining three studies, representatives of both sexes were recruited [16,19,20].

Most RCTs were conducted in the USA [15,17,18,19]. Two trials were conducted in Europe: one in Finland [14] and another in the UK [16]. One trial was conducted in China [20].

In two trials, the participants were current or former smokers [13,18], while the remaining RCTs had varying shares of current smokers, former smokers, and never smokers [15,16,17,18,19,20].

### 4.2. Summary of Conducted Meta-Analyses

**Figure 3:**
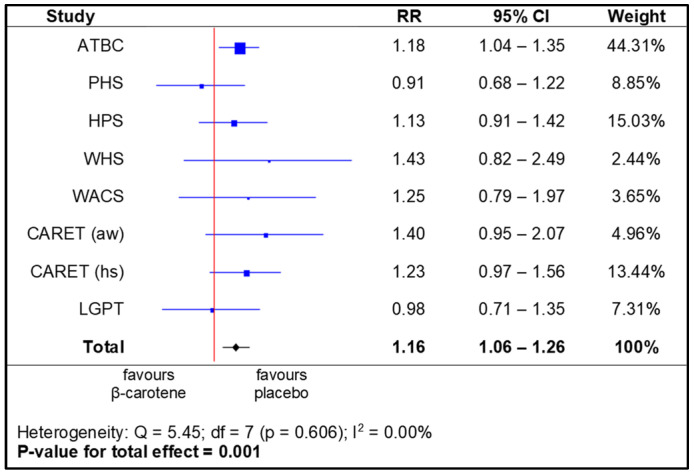
Meta-analysis in total—lung cancer incidence depending on beta-carotene supplementation (legend: ATBC—Alpha-Tocopherol, Beta-Carotene Cancer Prevention Study; PHS—Physicians’ Health Study; HPS—Heart Protection Study; WHS—Women’s Health Study; WACS—Women’s Antioxidant Cardiovascular Study; CARET (aw)—Carotene and Retinol Efficacy Trial, asbestos-workers; CARET (hs)—Carotene and Retinol Efficacy Trial, heavy smokers; LGPT—Linxian General Population Trial).

The meta-analysis of the data from all the studies gave an overall increased RR of 1.16 (95% CI), ranging from 1.06 to 1.26, in the group supplemented with beta-carotene compared to the placebo. This result was statistically significant, with *p* = 0.001 (Figure 3).

**Figure 4:**
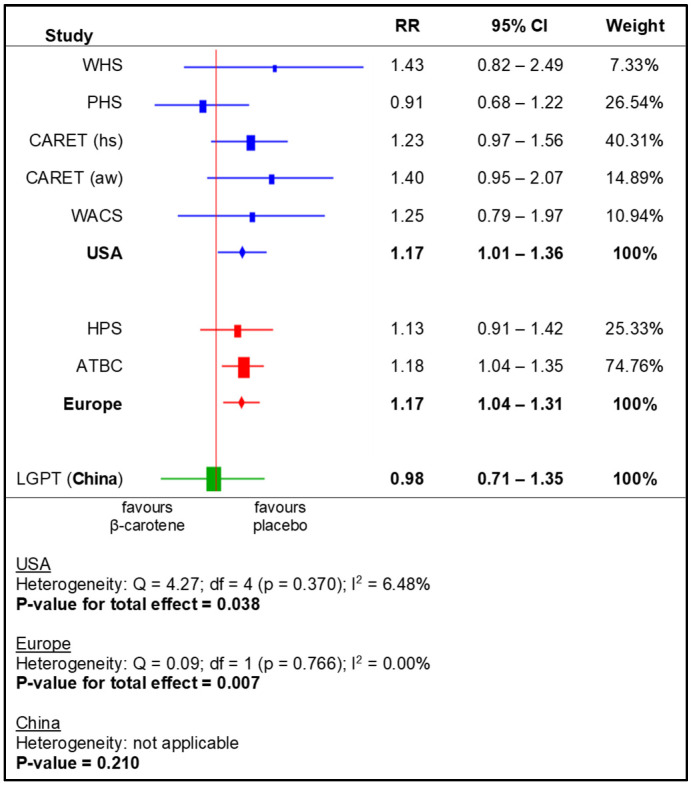
Meta-analysis for subgroups (world regions)—lung cancer incidence depending on beta-carotene supplementation (legend: WHS—Women’s Health Study; PHS—Physicians’ Health Study; CARET (aw)—Carotene and Retinol Efficacy Trial, asbestos-workers; CARET (hs)—Carotene and Retinol Efficacy Trial, heavy smokers; WACS—Women’s Antioxidant Cardiovascular Study; HPS—Heart Protection Study; ATBC—Alpha-Tocopherol, Beta-Carotene Cancer Prevention Study; LGPT—Linxian General Population Trial).

**Figure 5:**
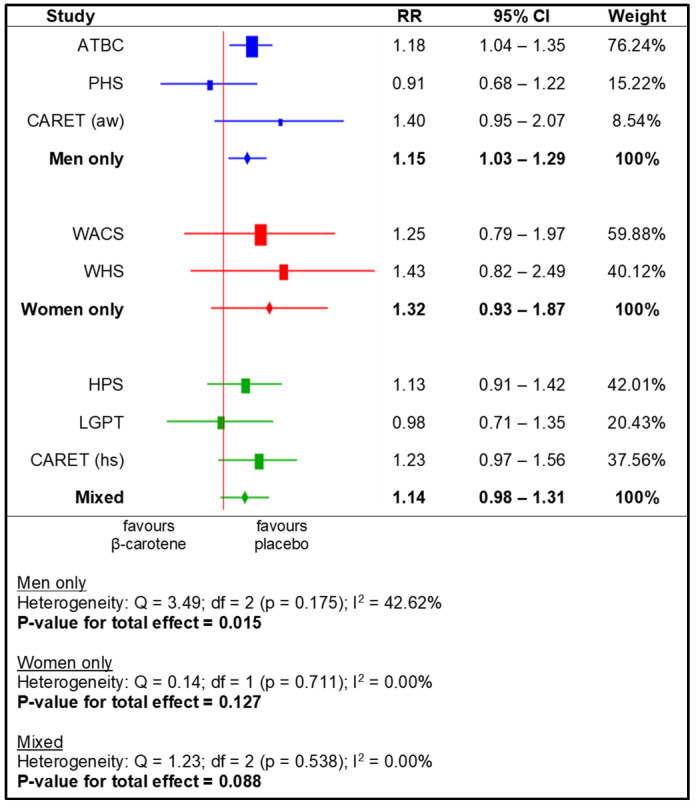
Meta-analysis for subgroups (sex)—lung cancer incidence depending on beta-carotene supplementation (legend: ATBC—Alpha-Tocopherol, Beta-Carotene Cancer Prevention Study; PHS—Physicians’ Health Study; CARET (aw)—Carotene and Retinol Efficacy Trial, asbestos-workers; WACS—Women’s Antioxidant Cardiovascular Study; WHS—Women’s Health Study; HPS—Heart Protection Study; LGPT—Linxian General Population Trial; CARET (hs)—Carotene and Retinol Efficacy Trial, heavy smokers).

**Figure 6:**
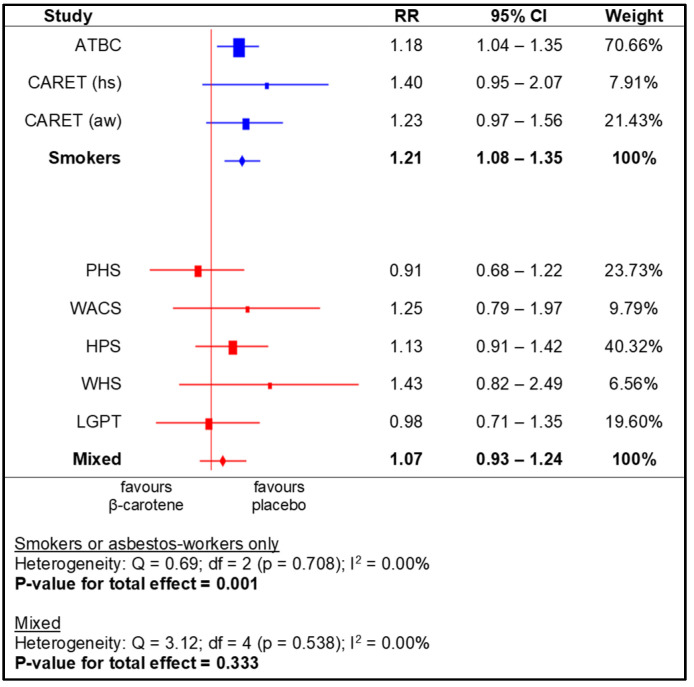
Meta-analysis for subgroups (smokers and asbestos-workers)—lung cancer incidence depending on beta-carotene supplementation (legend: ATBC—Alpha-Tocopherol, Beta-Carotene Cancer Prevention Study; CARET (hs)—Carotene and Retinol Efficacy Trial, heavy smokers; CARET (aw)—Carotene and Retinol Efficacy Trial, asbestos-workers; PHS—Physicians’ Health Study; WACS—Women’s Antioxidant Cardiovascular Study; HPS—Heart Protection Study; WHS—Women’s Health Study; LGPT—Linxian General Population Trial).

**Figure 7:**
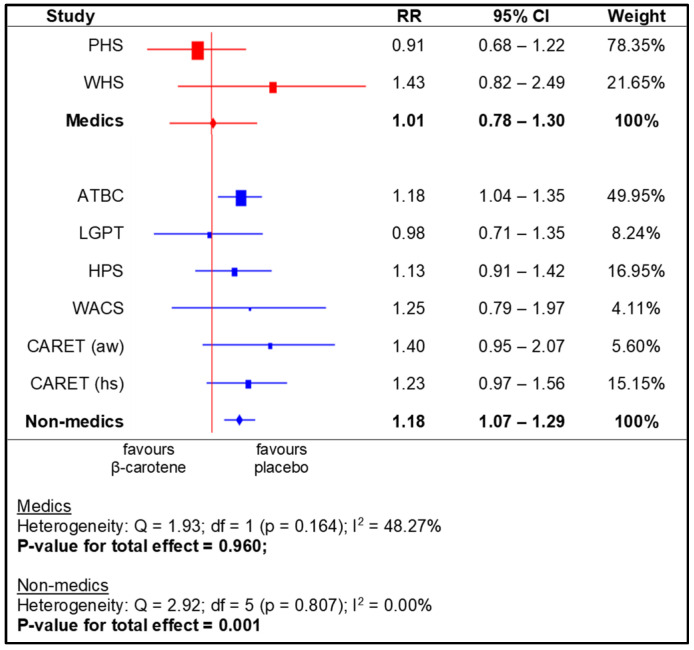
Meta-analysis for subgroups (medical professionals)—lung cancer incidence depending on beta-carotene supplementation (legend: PHS—Physicians’ Health Study; WHS—Women’s Health Study; ATBC—Alpha-Tocopherol, Beta-Carotene Cancer Prevention Study; LGPT—Linxian General Population Trial; HPS—Heart Protection Study; WACS—Women’s Antioxidant Cardiovascular Study; CARET (aw)—Carotene and Retinol Efficacy Trial, asbestos workers; CARET (hs)—Carotene and Retinol Efficacy Trial, heavy smokers).

The meta-analysis for the subgroups yielded a significantly higher RR for the following: US and European populations (i.e., based on region), men, smokers, and non-health professionals. No statistically significant effects of beta-carotene supplementation were observed in the other subgroups (Figure 4, Figure 5, Figure 6 and Figure 7).

### 4.3. Summary of Conducted Meta-Regression

**Figure 8:**
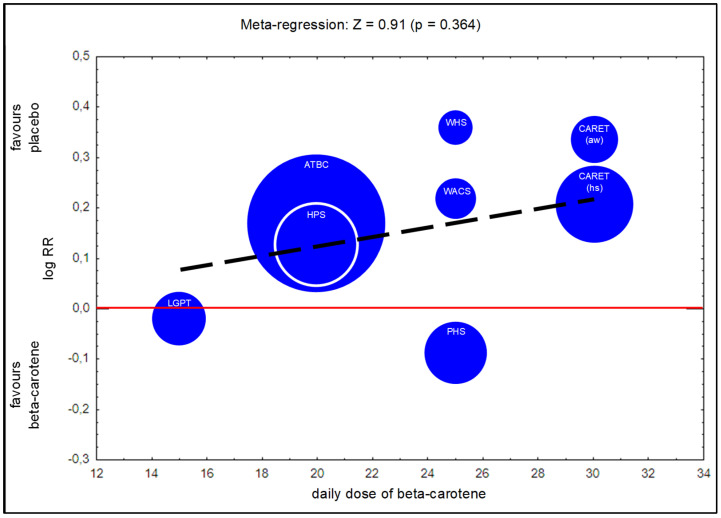
Meta-regression—lung cancer incidence depending on a daily dose of beta-carotene supplementation (legend: ATBC—Alpha-Tocopherol, Beta-Carotene Cancer Prevention Study; PHS—Physicians’ Health Study; HPS—Heart Protection Study; WHS—Women’s Health Study; WACS—Women’s Antioxidant Cardiovascular Study; CARET (aw)—Carotene and Retinol Efficacy Trial, asbestos-workers; CARET (hs)—Carotene and Retinol Efficacy Trial, heavy smokers; LGPT—Linxian General Population Trial).

The meta-regression analysis indicated no significant association between the size of the beta-carotene supplementation dose and increasing incidence of lung cancer (Figure 8).

## 5. Discussion

The conducted meta-analyses indicated that beta-carotene supplementation had no protective effect against lung cancer development. Moreover, the use of beta-carotene in the primary chemoprevention of lung cancer may increase the risk of lung cancer in some groups of people. These findings are in line with those obtained in a similar systematic review published a decade ago regarding beta-carotene supplementation in the primary prevention of various cancers [12].

Analysing the RCTs by location, in most cases, similar results were obtained from each subgroup as for the group as a whole. Only one trial conducted in China [20] suggested that beta-carotene supplementation had no effect on lung cancer risk. However, this study included a nutrient-inadequate population. The participants had a lower baseline level of beta-carotene in the plasma, so supplementation with a small dose of beta-carotene (15 mg—the lowest dose used in the studies included in the review) may not have significantly influenced the carcinogenesis process.

Regarding the sex of the participants, beta-carotene supplementation was observed to have a significant negative effect in the RCTs with only male participants. This observation supports those obtained in a previous meta-analysis [12] based on trials “with a majority of men”. The reason for such an observation may be the fact that lung cancer is generally more common in men than in women [3], which is most likely due to the higher prevalence of cigarette smoking in men than women [21].

Previous systematic reviews with meta-analyses have also found supplementation of beta-carotene to be associated with a significantly increased risk of lung cancer in groups of smokers and asbestos workers [12,22]. This fact was also highlighted by the US Center for Science in the Public Interest in a 2007 official letter to the Food and Drug Administration: these groups of people should not use any beta-carotene supplementation [23]. Our meta-analysis supports this. It may be explained by the fact that carotenoids, paradoxically, may increase the oxidative stress induced by tobacco smoke. Beta-carotene can readily form pro-oxidative oxidation products, especially at high concentrations in the oxidative environment of smokers’ lungs, characterized by increased cell oxidative stress and decreased antioxidant defence. Antioxidant supplements may alter the redox balance with deleterious effects on cellular functions, supporting cancerogenesis [24].

With regard to profession, i.e., medical professionals vs. non-medics, it was found that beta-carotene supplementation had no effect among the former group and a negative effect in the latter. This may be due to medical professionals (e.g., doctors, nurses) having a greater awareness of a healthy lifestyle, which may have protective effects on lung cancer.

A meta-analysis conducted by Druesne-Pecollo et al. [12] also indicated an increased risk of lung cancer in trials where the daily dose of beta-carotene supplementation was ≥20 mg (RR = 1.16, 95% CI = 1.06–1.27) vs. lower doses (RR = 0.93, 95% CI = 0.69–1.25). Therefore, in the present study, the relationship between the dose of beta-carotene supplementation and the magnitude of the negative effect associated with lung cancer risk was tested using meta-regression. The result revealed no statistically significant association, not explaining the studied variability; however, a general trend was observed, suggesting that the risk of lung cancer increases with daily beta-carotene dose. Additional research is required to confirm whether the beta-carotene supplementation dose has a more substantial effect on lung cancer risk.

## 6. Conclusions

Beta-carotene supplementation clearly has a negative effect on the risk of lung cancer in smokers and asbestos workers. However, further RCTs are needed to clarify some issues raised by our findings. For example, most of the trials included in this review were conducted in the USA, and further studies are, hence, needed in Europe and Asia, particularly in the latter as only one trial has been performed in this region to date. Additionally, research involving non-smokers and people free from exposure to dust (e.g., asbestos) would be especially valuable.

The meta-regression results did not indicate that the dose of beta-carotene supplementation had any statistically significant effect on the change in the degree of lung cancer risk. However, a noticeable trend suggests that the risk of lung cancer increases with increasing beta-carotene intake. This point could be clarified by additional RCTs performed on supplementation with different doses of beta-carotene.

Moreover, additional RCTs are needed on the effect of astaxanthin supplementation on cancer risk as there are no reports on this subject in the available literature. Astaxanthin demonstrates far greater antioxidant potential than beta-carotene, offering promising prospects for cancer chemoprevention.

## Figures and Tables

**Table 1:** Examples of carotenes and xanthophylls.

Carotenes	Xanthophylls
beta-carotene	lutein
alpha-carotene	astaxanthin
lycopene	zeaxanthin
beta-Apo-8′-carotenal	canthaxanthin

**Table 2:** Characteristics of included studies for systematic review.

Study	Participants						Follow-Up [yrs]	Intervention		RR (95% CI)	Ref.
	Total [No]	Description	Country	Age (Mean) [y]	Male/Female [%]	Smokers Current/Former [%]		Beta-Carotene Dose	Other Substances		
ATBC	29,133	smokers	Finland	50–69 (57.2)	100/0	100/0	5–8; median 6.1	20 mg daily	50 mg α-toco-ferol or none	1.18 (1.04–1.35)	[14]
PHS	22,071	physicians	USA	40–84 (53.0)	100/0	39/11	11.7–14.4; mean 12.9	50 mg on alternate days	325 mg aspirin	0.91 (0.68–1.22)	[15]
HPS	20,536	patients with cardiac risk factors	UK	40–80 (ND)	75.3/24.7	14/61	mean 5	20 mg daily	600 mg vitamin E and 250 mg vitamin C	1.13 (0.91–1.42)	[16]
WHS	39,876	health professionals	USA	45+ (ND)	0/100	13/ND	0–2.7; median 2.1	50 mg on alternate days	600 IU vitamin E and/or100 mg aspirin or none	1.43 (0.82–2.49)	[17]
WACS	7627	patients with cardiac risk factors	USA	40+ (60.4)	0/100	15/42	mean 9.4	50 mg on alternate days	500 mg vitamin C and/or 600 IU vitamin E or none	1.25 (0.79–1.97)	[18]
CARET	4060	asbestos-workers	USA	45–74 (57.7)	100/0	39/58	0–5.5; mean 4	30 mg daily	25,000 IU retinol	1.40 (0.95–2.07)	[19]
CARET	14,254	heavy smokers	USA	45–69 (58.5)	55.9/44.1	66/34	0–5.5; mean 4	30 mg daily	25,000 IU retinol	1.23 (0.97–1.56)	[19]
LGPT	29,584	nutrient-inadequate population	China	40–69 (ND)	44.8/55.2	30 *	0–10	15 mg daily	50 µg selenium and 30 mg vitamin E	0.98 (0.96–1.00)	[20]

Legend: ATBC—Alpha-Tocopherol, Beta-Carotene Cancer Prevention Study; PHS—Physicians’ Health Study; HPS—Heart Protection Study; WHS—Women’s Health Study; WACS—Women’s Antioxidant Cardiovascular Study; CARET—Carotene and Retinol Efficacy Trial; LGPT—Linxian General Population Trial; RR—relative risk; CI—confidence intervals; Ref.—references; ND—no data; *—ever smoked for minimum six years.

## Data Availability

Not applicable.
